# Insulin-Like Growth Factor 2 mRNA Binding Protein 3 Promotes Cell Proliferation of Malignant Mesothelioma Cells by Downregulating p27^Kip1^

**DOI:** 10.3389/fonc.2021.795467

**Published:** 2022-01-19

**Authors:** Ihiro Endo, Vishwa Jeet Amatya, Kei Kushitani, Takahiro Kambara, Tetsuya Nakagiri, Yutaro Fujii, Yukio Takeshima

**Affiliations:** Department of Pathology, Hiroshima University Graduate School of Biomedical and Health Sciences, Hiroshima, Japan

**Keywords:** mesothelioma, IGF2BP3, siRNA, p27, cell line, proliferation

## Abstract

Malignant mesothelioma is a tumor with a poor prognosis, mainly caused by asbestos exposure and with no adequate treatment yet. To develop future therapeutic targets, we analyzed the microarray dataset GSE 29370 of malignant mesothelioma and reactive mesothelial hyperplasia, downloaded from the Gene Expression Omnibus (GEO) database. We identified insulin-like growth factor 2 mRNA binding protein 3 (IGF2BP3) as one of the significantly upregulated genes in malignant mesothelioma. IGF2BP3 functions as an oncoprotein in many human cancers; however, to our knowledge, this is the first study on the biological function of IGF2BP3 in malignant mesothelioma cells. The knockdown of IGF2BP3 in malignant mesothelioma cells resulted in the suppression of cell proliferation with an increase in the proportion of cells in the G1 phase of the cell cycle. Furthermore, knockdown of IGF2BP3 inhibited cell migration and invasion. We focused on the cell cycle assay to investigate the role of IGF2BP3 in cell proliferation in malignant mesothelioma. Among the various proteins involved in cell cycle regulation, the expression of p27 Kip1 (p27) increased significantly upon IGF2BP3 knockdown. Next, p27 siRNA was added to suppress the increased expression of p27. The results showed that p27 knockdown attenuated the effects of IGF2BP3 knockdown on cell proliferation and G1 phase arrest. In conclusion, we found that IGF2BP3 promotes cell proliferation, a critical step in tumorigenesis, by suppressing the expression of p27 in malignant mesothelioma.

## Introduction

Malignant mesothelioma is a highly aggressive tumor that is mainly caused by occupational and environmental exposure to asbestos. The incidence of malignant mesothelioma is increasing worldwide, especially in developing countries, and the mortality rate in Japan is expected to peak by 2030 (1, 2). Although multidisciplinary therapies, including surgery, chemotherapy, and radiation therapy, are used to treat malignant mesothelioma, sufficiently effective treatments have not yet been established (3, 4). The molecular mechanisms of mesothelioma carcinogenesis are not fully understood, and it is important to evaluate potential new therapeutic targets for the development of new therapies.

Insulin-like growth factor 2 mRNA-binding protein 3 (IGF2BP3) is a member of the IGF2BP family (5) and plays a vital role in the transport, stabilization, and translational regulation of multiple mRNAs (6, 7). IGF2BP3, an oncofetal protein, is not expressed in normal adult tissues (8). IGF2BP3 is highly expressed in human cancers, including lung cancer (9), melanoma (10), colorectal cancer (11), liver cancer (12), and squamous cell carcinoma of the head and neck (13). The expression of IGF2BP3 has a significant influence on biological functions related to tumorigenesis, such as cell proliferation and migration in various human cancers, including esophageal cancer (14), breast cancer (15), colorectal cancer (16), and prostate cancer (17). IGF2BP3 also contributes to tumorigenesis in many human organs and its malignant progression.

There are a few studies on IGF2BP3 expression in malignant mesothelioma. IGF2BP3 has been reported as a prognostic biomarker of malignant mesothelioma, and its reduced expression has a positive effect on life expectancy (18). Furthermore, IGF2BP3 has been used as a differential diagnostic marker to distinguish malignant mesothelioma from reactive mesothelial hyperplasia (19–21). These studies suggest that IGF2BP3 has diagnostic and prognostic value in malignant mesothelioma. However, biological studies have not yet been conducted. In this study, we conducted a functional analysis of IGF2BP3 in malignant mesothelioma cell lines and the regulation of downstream genes.

## Materials And Methods

### Analysis of Gene Expression Data

Microarray data were downloaded from GEO datasets. The GSE 29370 (22) was used to analyze 11 malignant mesothelioma and 2 reactive mesothelioma hyperplasia samples; the differentially expressed genes were examined based on those that showed more than 1.5-fold change, using the Subio Platform software (Subio, Amami-shi, Japan). The histological types of 11 malignant mesothelioma samples used were as follows: MM26-P (epithelioid), MM34-P (epithelioid), MM35-P (epithelioid), MM45-P (epithelioid), MM46-P (sarcomatoid), MM16-P (biphasic), MM30-P (biphasic), H28 (epithelioid), H2452 (epithelioid), HMMME (epithelioid), MSTO-211H (biphasic).

### Mesothelioma Cell Lines

Two human mesothelioma cell lines were used in this study. The ACC-MESO1 mesothelioma cell line was purchased from RIKEN BioResource Research Center (Tsukuba, Japan), and the CRL-5915 mesothelioma cell line was purchased from the American Type Culture Collection (Manassas, VA, USA). The cell lines used were sampled from malignant pleural mesothelioma, and the histological types are ACC-MESO1 for fibroblast-like type and CRL-5915 for epithelioid type. Cells were cultured in Roswell Park Memorial Institute 1640 medium (RPMI-1640) supplemented with kanamycin, amphotericin B, and 5% fetal bovine serum (Thermo Fisher Scientific, Tokyo, Japan). Cells were maintained in culture dishes at 37°C in a humidified incubator with 5% CO_2_.

### Transfection of Mesothelioma Cells

IGF2BP3 siRNA (#s20919), p27 Kip1 siRNA (#s2837), and negative control (NC) siRNA were purchased from Thermo Fisher Scientific (Tokyo, Japan). Mesothelioma cells at 60%–80% confluence were transfected with IGF2BP3, p27, or NC siRNA using Lipofectamine RNAiMAX (Thermo Fisher Scientific) in Opti-MEM Reduced Serum Medium (Thermo Fisher Scientific) according to the manufacturer’s recommended protocols.

### Quantitative Reverse-Transcription Polymerase Chain Reaction

Mesothelioma cell lines (3 × 10^5^) were transfected with 25 pmol of IGF2BP3 or NC siRNA in 6-well plates for 72 h. RNA was extracted from the cells using Maxwell RSC RNA Cells Kits using the Maxwell RSC Instrument (Promega KK, Tokyo, Japan) according to the manufacturer’s protocols. The extracted RNA was reverse transcribed with SuperScript IV VILO Master Mix (Thermo Fisher Scientific) and amplified using the PowerUp SYBR Green Master Mix (Thermo Fisher Scientific) on an AriaMax Real-Time PCR System (Agilent Technologies, Tokyo, Japan). The relative expression levels were calculated using the comparative Cq method. Expression levels were normalized to that of glyceraldehyde 3-phosphate dehydrogenase (GAPDH). The primer sequences used for quantitative reverse-transcription polymerase chain reaction (qRT-PCR) were as follows:

IGF2BP3 forward: 5′-AGT TGT TGT CCC TCG TGA CC-3′

IGF2BP3 reverse: 5′-GTC CAC TTT GCA GAG CCT TC-3′

GAPDH forward: 5′-ACA ACT TTG GTA TCG TGG AAG G-3′

GAPDH reverse: 5′-GCC ATC ACG CCA CAG TTT C-3′

### Cell Proliferation Assay

Mesothelioma cell lines (3 × 10^3^ cells) were incubated with 1 pmol IGF2BP3 siRNA, p27 siRNA, or NC siRNA in 96-well plates for 72 or 96 h. The proliferation rate was determined at 24, 48, 72, and 96 h using the Cell Titer Glo 2.0 reagent (Promega) and the GloMax Explorer microplate reader (Promega) according to the manufacturer’s recommended protocols, by measuring the ATP level relative to the number of viable cells.

### Cell Cycle Assay

Mesothelioma cell lines (1 × 10^5^ cells) were transfected with 5 pmol IGF2BP3, p27, or NC siRNA in 24-well plates for 72 h, following which the cells were collected and fixed in 70% ethanol in 15 mL centrifuge tubes for approximately 1 h. After centrifugation and ethanol removal, the Guava Cell Cycle Reagent (Luminex, Austin, TX, USA) was added. This reagent contains propidium iodide, which allows for discrimination between cells at different stages of the cell cycle by labeling the cellular DNA. The signal intensity of the DNA labelling was measured using a Guava EasyCyte Mini flow cytometer (Guava Technologies, Hayward, CA, USA) according to the manufacturer’s protocol. Cell cycle data were analyzed using FCS Express 5.0 (DeNovo, Los Angeles, USA).

### Cell Migration Assay

Mesothelioma cell lines were incubated overnight with 5 pmol IGF2BP3, p27, or NC siRNAs in collagen-coated 24-well plates. A wound was created by scratching each well with a 1 mL micropipette tip. The wells were washed twice to remove floating cells and subsequently incubated. The gap area (wound) was photographed every 12 h using a CKX53 inverted microscope equipped with a DP21 digital camera (Olympus, Tokyo, Japan), and the gap area was further analyzed using ImageJ software (https://imagej.nih.gov/ij/index.html).

### Cell Invasion Assay

ACC-MESO1 (1 × 10^5^ cells) and CRL-5915 (3 × 10^5^ cells) were incubated with 5 pmol siRNA in BD FluoroBlok culture inserts (BD Biosciences, Franklin Lakes, NJ, USA) with 8 μm pores. The ACC-MESO1 and CRL-5915 cells were analyzed 48 h and 72 h after transfection, respectively. Infiltrating cells were stained with Hoechst 33342 (Thermo Fisher Scientific) for 10 min, and images of the infiltrating cells were acquired using an IX81 inverted fluorescence microscope equipped with a DP80 digital camera (Olympus, Tokyo, Japan). Fluorescence images were analyzed using ImageJ software (https://imagej.nih.gov/ij/index.html), and the total number of infiltrating cells was determined.

### Western Blot Analysis

Mesothelioma cell lines (3 × 10^5^) were transfected with 25 pmol IGF2BP3, p27, or NC siRNA in 6-well plates for 72 h. Cell lysates were prepared using the RIPA Lysis Buffer System (Santa Cruz Biotechnologies, Dallas, TX, USA), and the total protein was quantified using a Qubit Fluorometer (Thermo Fisher Scientific). The total protein (20 μg) was separated on a Bolt 4%–12% Bis-Tris Plus Gel (Thermo Fisher Scientific) with lithium dodecyl sulfate (LDS) electrophoresis at 165 V for 35 min. It was then transferred onto a polyvinylidene difluoride (PVDF) membrane using a Mini Blot Module (Thermo Fisher Scientific) at 20 V for 60 min. After blocking with 5% bovine serum albumin in TBS-T, the membranes were incubated overnight with primary antibodies. Anti-IGF2BP3 antibody (1:2000, polyclonal, #14642-1-AP) was purchased from Proteintech (Rosemont, IL, USA). p27 Kip1(1:3000, #3686), CDK2 (1:3000, #2546), cyclin E1 (1:3000, #20808), phospho-RB (1:3000, #8516), and GAPDH (1:5000, #2118) were purchased from Cell Signaling Technology (Danvers, MA, USA). The membrane was then incubated with secondary antibody. The secondary antibody used was anti-rabbit IgG-HRP (1:2000, #7074, Cell Signaling Technology). The membrane was stained with ImmunoStar LD (Wako Pure Chemical Industries, Osaka, Japan) and captured using a c-Digit Blot Scanner (LICOR, Lincoln, NE, USA).

### Statistical Analysis

The experiments were performed in triplicate, and the data are expressed as the mean ± the standard deviation. The difference between the two groups was analyzed using an unpaired Student’s t-test. The statistical significance was set at *p* < 0.05.

## Results

### IGF2BP3 Is Upregulated in Malignant Mesothelioma Tissue and Cell Lines

**Figure 1:**
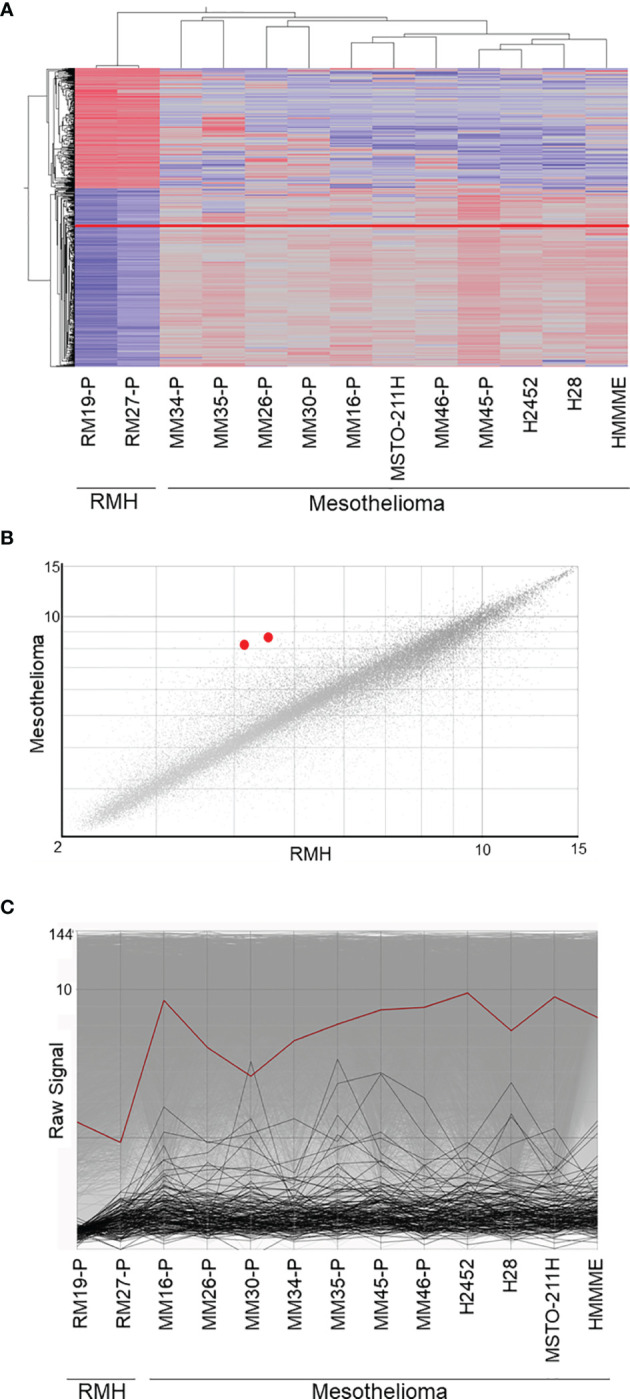
Microarray data analysis. **(A)** Supervised hierarchical clustering of 11 malignant mesothelioma samples and 2 reactive mesothelial hyperplasia samples. IGF2BP3, presented as a red line, is upregulated in malignant mesothelioma, as compared with its expression in reactive mesothelioma hyperplasia. **(B)** The scatter plot showing the correlation between gene expression in malignant mesothelioma and reactive mesothelial hyperplasia. The two IGF2BP3 transcripts, shown as red dots, were upregulated in malignant mesothelioma when compared against expression in reactive mesothelial hyperplasia. **(C)** Line graph showing the gene expression in malignant mesothelioma and reactive mesothelial hyperplasia; IGF2BP3 expression, represented by the red line, is higher in malignant mesothelioma than in reactive mesothelial hyperplasia. RMH, reactive mesothelial hyperplasia.

Using the GSE29370 dataset from the GEO database, we analyzed 11 malignant mesothelioma samples and 2 reactive mesothelial hyperplasia samples. We identified 378 upregulated transcripts and 256 downregulated transcripts with more than 1.5-fold change in malignant mesothelioma compared to those in reactive mesothelial hyperplasia, as shown by the hierarchical clustering (**Figure 1A**). The mean transcript level of IGF2BP3 in mesothelioma increased by approximately 2-fold as shown in the scatter plot (**Figure 1B**). All seven mesothelioma tissues and four mesothelioma cell lines showed high expression of IGF2BP3 compared with two reactive mesothelial hyperplasia tissues (**Figure 1C**).

### IGF2BP3 Knockdown Reduces Proliferation With G1 Phase Arrest, Migration, and Invasion of Mesothelioma Cell

**Figure 2:**
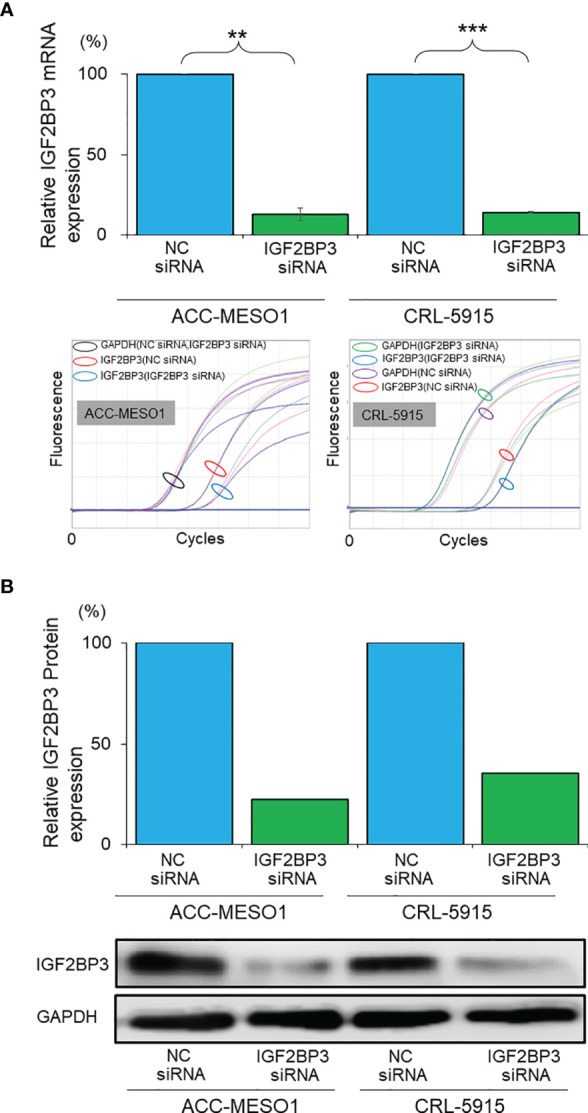
Validation of IGF2BP3 knockdown. **(A)** Bar graph showing the relative IGF2BP3 mRNA expression in mesothelioma cell lines transfected with IGF2BP3 siRNA as compared with those transfected with NC siRNA (the lower panel represents the amplification curves of real time PCR experiments). **(B)** Bar graph showing the relative IGF2BP3 protein expression in mesothelioma cell lines transfected with IGF2BP3 siRNA as compared with those transfected with the NC siRNA (lower panel is an image of the western blot). NC, negative control; **p < 0.01; ***p < 0.001.

IGF2BP3 siRNA was transfected into malignant mesothelioma cells to knock down IGF2BP3, and the efficiency of transfection was evaluated by western blotting and RT-PCR. IGF2BP3 mRNA expression was suppressed by 86.9% in ACC-MESO1 cells and 85.7% in CRL-5915 cells transfected with IGF2BP3 siRNA, as compared with those transfected with NC siRNA (**Figure 2A**). Furthermore, IGF2BP3 protein expression was suppressed by 77.4% in ACC-MESO1 and by 64.5% in CRL-5915, compared with that in the negative control (**Figure 2B**).

**Figure 3:**
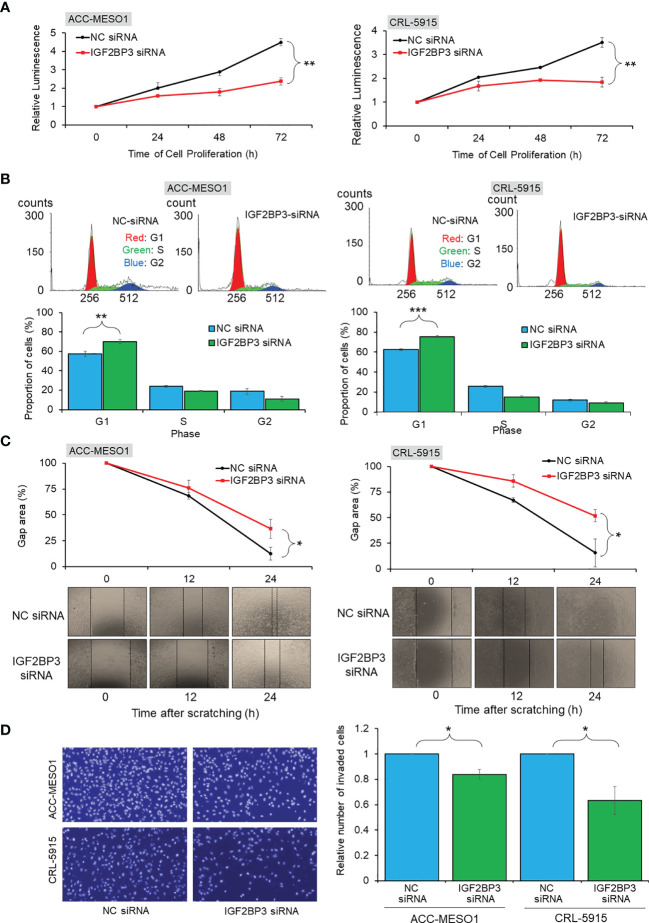
Functional assays of IGF2BP3 expression. **(A)** Cell proliferation assay. Line chart showing reduced cell proliferation in ACC-MESO1 and CRL-5915 cells with IGF2BP3 siRNA transfection, compared with those cells transfected with NC siRNA. **(B)**Cell cycle assay. Cell cycle histogram and bar graph showing the G1 arrest of ACC-MESO1 and CRL-5915 by IGF2BP3 siRNA transfection. **(C)** Migration assay. Line chart and images showing reduced wound gap area of ACC-MESO1 and CRL-5915 following IGF2BP3 siRNA transfection. **(D)** Invasion assay. Bar graph and images showing reduced numbers of invading ACC-MESO1 and CRL-5915 cells, following transfection with IGF2BP3 siRNA. NC, negative control; NS, no significance; *p < 0.05; **p < 0.01; ***p < 0.001.

IGF2BP3 siRNA transfection suppressed the proliferation of ACC-MESO1 cells by 47.2% and CRL-5915 cells by 47.5% after 72 h, when compared with that of cells transfected with NC siRNA (**Figure 3A**). Given that the IGF2BP3 knockdown suppressed cell proliferation, we further investigated the effect of IGF2BP3 on cell cycle progression. The proportion of ACC-MESO1 and CRL-5915 cells in the G1 phase of the cell cycle was higher with IGF2BP3 siRNA transfection (69.9% and 75.4%, respectively) than with NC siRNA transfection (57.3% and 62.5%, respectively) (**Figure 3B**).

IGF2BP3 siRNA transfection decreased the wound gap area more slowly than NC siRNA transfection in both mesothelial cell lines. Transfection with IGF2BP3 siRNA significantly reduced the migration of both ACC-MESO1 and CRL-5915 cells, by 23.9% and 36.0%, respectively, as compared with that by NC siRNA transfection (**Figure 3C**).

IGF2BP3 knockdown significantly reduced cell invasion in both mesothelial cell lines Transfection with IGF2BP3 siRNA reduced the number of invading cells by 16.0% in ACC-MESO1 cells and 36.5% in CRL-5915 cells as compared with the cell numbers after NC siRNA transfection (**Figure 3D**).

### IGF2BP3 Knockdown Regulates p27, CDK2, Cyclin E1, and Phospho-RB

**Figure 4:**
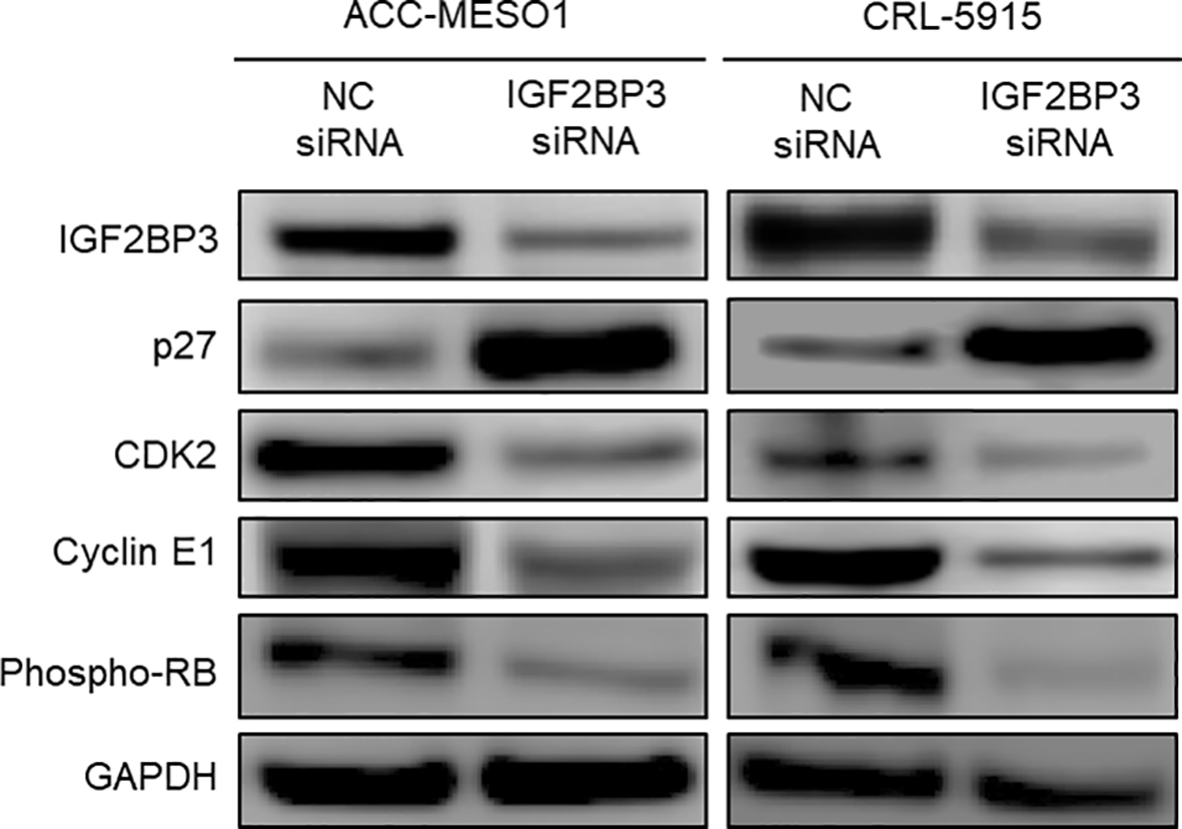
Downstream regulation of IGF2BP3. Western blots of IGF2BP3, p27, CDK2, Cyclin E1, and phospho-RB proteins in ACC-MESO1 and CRL-5915 cells show increased expression of p27 and decreased expression of CDK2, Cyclin E1, and phospho-RB with IGF2BP3 siRNA transfection. NC: negative control.

By comparing IGF2BP3 siRNA-transfected cells with NC siRNA-transfected cells, we comprehensively examined the differential expression of proteins related to cell cycle regulation by western blotting. IGF2BP3 siRNA transfection significantly increased the expression of p27 and decreased the expression of CDK2, cyclin E1, and phospho-RB in both mesothelial cell lines compared to those after NC siRNA transfection (**Figure 4**). IGF2BP3 knockdown in ACC-MESO1 cells increased p27 protein expression by 424.2% and decreased the expression of CDK2 protein by 52.7%, cyclin E1 by 35.4%, and phospho-RB by 60.5%. The changes in protein expression of CRL-5915 were as follows: p27, 508.2%, CDK2, 65.4%; cyclin E1, 83.5%; and phospho-RB, 86.3%.

### IGF2BP3 Increased Cell Proliferation by Suppressing p27

**Figure 5:**
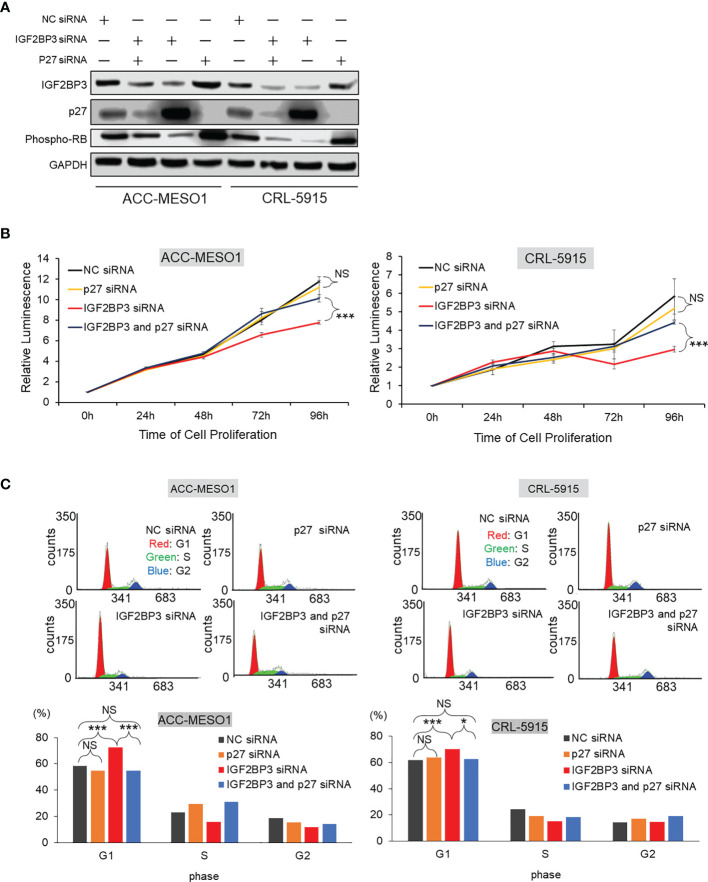
Effect of IGF2BP3 and p27 on cell proliferation and G1 cell cycle phase arrest. **(A)** Western blot of IGF2BP3, p27, and phospho-RB proteins. IGF2BP3 or p27 siRNA- transfected cells show the suppression of specific protein expression in ACC-MESO1 and CRL-5915 cells. Cell lines transfected simultaneously with both IGF2BP3 and p27 siRNAs re-diminish p27 protein expression and recover phospho-RB protein expression. **(B)** Cell proliferation assay. In ACC-MESO1 and CRL-5915, transfection with IGF2BP3 siRNA and p27 siRNA together show a significant increase in cell proliferation at 72 h and 96 h from transfection of IGF2BP3 siRNA alone. Note the cells transfected with p27 siRNA alone show no significant change from those transfected with NC siRNA. **(C)** Cell cycle assay. ACC-MESO1 and CRL-5915 cells transfected with IGF2BP3 siRNA and p27 siRNA together, show significant reduction in the percentage of cells in G1 phase of the cell cycle. Note the cells transfected with p27 siRNA alone show no significant difference in the percentage of cells in G1 phase compared with those cells transfected with NC siRNA. NC, Negative control; NS, no significance; *p < 0.05; ***p < 0.001.

To investigate whether IGF2BP3 causes cell proliferation by suppressing p27, mesothelioma cells were transfected with either one or both IGF2BP3 siRNA and p27 siRNA. Western blotting showed the suppression of specific protein expression in IGF2BP3 or p27 siRNA-transfected cells (**Figure 5A**). Furthermore, when mesothelial cells were transfected with both IGF2BP3 and p27 siRNA, elevated p27 protein expression by IGF2BP3 knockdown was diminished, and repressed phospho-RB protein was recovered.

Mesothelioma cells simultaneously transfected with IGF2BP3 siRNA and p27 siRNA showed a significant recovery in cell proliferation, when compared with cells transfected with IGF2BP3 siRNA alone. Specifically, in ACC-MESO1 cells, transfection with IGF2BP3 siRNA and p27 siRNA together increased cell proliferation; cells were initially transfected with IGF2BP3 siRNA alone, then transfected with the combination of IGF2BP3 and p27 siRNAs after 72 h and 96 h, resulting in increases in cell proliferation of 31.8% and 30.4%, respectively. Similarly, when IGF2BP3 siRNA and p27 siRNA were transfected together in CRL-5915 cells, there was a 43.8% increase in cell proliferation with addition 72 h after transfection of IGF2BP3 siRNA alone, and a 48.8% increase with addition after 96 h. In contrast, mesothelial cells transfected with p27 siRNA alone showed no significant change in cell proliferation compared with that of cells transfected with NC siRNA (**Figure 5B**). These results indicate that the repression of p27 expression is significant for IGF2BP3 to activate cell proliferation in mesothelioma cells.

Furthermore, we examined the effects of IGF2BP3 and p27 on cell cycle progression. When p27 siRNA and IGF2BP3 siRNA were simultaneously transfected into mesothelioma cells, there was an increase in the percentage of cells in the G1 phase of the cell cycle compared with the cell numbers after transfection with IGF2BP3 siRNA alone. Specifically, when IGF2BP3 siRNA and p27 siRNA were transfected together, the percentage of cells in the G1 phase was reduced by 18.0% in ACC-MESO1 cells and by 7.6% in CRL-5915 cells. However, when p27 siRNA alone was transfected into mesothelioma cells, there was no significant difference in the percentage of cells in the G1 phase compared with that after NC siRNA transfection (**Figure 5C**).

## Discussion

Malignant mesothelioma is a tumor with a poor prognosis that develops in mesothelial cells, primarily due to asbestos exposure. The current first-line treatment for malignant mesothelioma is a combination of cisplatin and pemetrexed (23), and very few patients undergo surgery (24). The survival rate for patients with malignant pleural mesothelioma is still low; a population-based study reported average survival times ranging from 5 months to 13.2 months (25). Although immunotherapeutic approaches to malignant mesothelioma have received much attention, further studies are needed to show a clear advantage over standard chemotherapy (26). A recent study of immunotherapeutic approach also showed significant and clinically meaningful improvements in overall survival versus standard chemotherapy (27). It is essential to develop novel treatments and to explore effective therapeutic targets against malignant mesothelioma.

In this study, we searched for genes that were significantly altered in malignant mesothelioma compared with those in reactive mesothelial hyperplasia to identify therapeutic targets. We analyzed 11 malignant mesothelioma cell samples and 2 reactive mesothelial hyperplasia samples from the microarray dataset GSE 29370. IGF2BP3 expression was significantly increased in mesothelioma cell samples compared with that in reactive mesothelial hyperplasia.

IGF2BP3 is an oncogenic protein that is overexpressed in many human cancers. IGF2BP3 functions as a biomarker of aggressiveness and metastasis in many tumors (28–31). IGF2BP3 expression correlates with poor prognosis in malignant mesothelioma (32). Structurally, IGF2BP3 has two N-terminal RNA recognition motifs (RRMs) and four C-terminal messenger ribonucleoprotein K homology (KH) domains (33). IGF2BP3 has been shown to target RNA as an RNA-binding protein and promote tumorigenesis primarily through regulation at the transcriptional level (34). IGF2BP3 was first identified for its ability to promote the translation of IGF2 mRNA (34). The currently-identified mRNA targets of IGF2BP3 include CD44 (6), MMP9 (15), HMGA2 (35), and PDPN (36). This is the first study to investigate the biological functions of IGF2BP3 in mesothelioma cells.

First, we performed IGF2BP3 knockdown in mesothelioma cell lines (ACC-MESO1 and CRL-5915) using siRNA to analyze its biological functions, including cell proliferation, cell cycle, cell migration, and cell invasion. IGF2BP3 knockdown suppressed cell proliferation and induced G1 cell cycle arrest in both cell lines. Furthermore, IGF2BP3 knockdown suppressed cell migration and invasion. These results suggest that IGF2BP3 strongly contributes to tumorigenesis in malignant mesothelioma.

**Figure 6:**
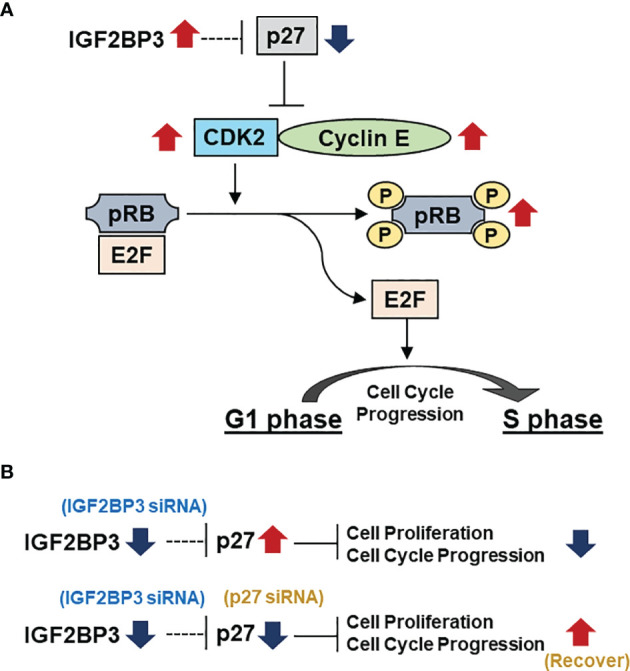
IGF2BP3 represses p27, thereby affecting the downstream pathway and finally causing G1 arrest. **(A)** IGF2BP3 activates the CDK2/Cyclin E complex by repressing p27, which leads to the phosphorylation of RB and the release of the transcription factor E2F from RB, leading to the progression from G1 to S phase in the cell cycle. **(B)**Transfection with IGF2BP3 siRNA alone results in inhibition of cell proliferation with G1 phase arrest by increasing the expression of p27. Simultaneous transfection with IGF2BP3 and p27 siRNA prevents p27 upregulation by IGF2BP3 siRNA, leading to alleviation of G1 phase arrest and restoration of cell proliferation.

The regulation of cell cycle proteins has long been an important field of oncology research (37). Cells proliferate successfully by passing through the G1, S, G2, and M phases of the cell cycle. A central component of the cell cycle regulatory system is cyclin-dependent kinase (CDK), which is regulated by cyclin binding, phosphorylation, and CDK inhibitors (37). A cell cycle assay revealed that IGF2BP3 knockdown induced G1 phase arrest. Therefore, we comprehensively examined the effect of IGF2BP3 knockdown on the expression of proteins related to cell cycle regulation. We found that IGF2BP3 knockdown significantly increased the expression of p27, followed by markedly decreasing CDK2 and cyclin E1 expression, and suppressing the phosphorylation of RB. Among the E2F family as transcriptional factors, E2F1, E2F2, and E2F3 were shown to be associated with RB in cell proliferation (38). In addition, E2F promotes transcription by targeting E2F itself and TYMS, POLA1, ORC1, FBXO5, and RRM2 as mentioned in G1/S- specific transcription pathway of Reactome website (https://reactome.org/PathwayBrowser/#/R-HSA-453279&SEL=R-HSA-69205&PATH=R-HSA-1640170,R-HSA-69278) as known targets of E2F, which are involved in DNA biosynthesis, regulation, and G1/S transition. Therefore, we investigated the expression of E2F1, E2F2, E2F3, and TYMS, POLA1, ORC1, FBXO5, and RRM2 as targets of E2F. The real time qPCR showed, in both ACC-MESO1 and CRL-5915 cell lines, the reduced expression of E2F family and E2F target genes by IGF2BP3 siRNA transfection compared to NC siRNA transfection (**Supplementary Figure**). The CDK inhibitor, p27, was first identified as a tumor suppressor protein able to induce G1 phase arrest (39); it binds to the CDK2/Cyclin E complex and inhibits its activity (40, 41). Overactivation of the cyclin CDK2/Cyclin E complex results in genomic instability and the development of tumors (42). Activation of the CDK2/Cyclin E complex induces RB protein phosphorylation and releases activated E2F, resulting in cell cycle progression from the G1 phase to S phase and cell proliferation (43–45). In summary, our results suggest that IGF2BP3 activates CDK2/Cyclin E1 and phosphorylates RB and activates E2F by suppressing the expression of p27, thereby facilitating the progression from the G1 phase of the cell cycle (**Figure 6A**).

We further verified that p27 suppression is a critical factor in IGF2BP3-induced cell proliferation. First, mesothelioma cells transfected with p27 siRNA alone did not show significant changes in cell proliferation compared with those transfected with NC siRNA. In mesothelioma cells without knockdown of IGF2BP3, the expression of p27 itself is usually a low level, suggesting that p27 knockdown alone did not cause significant changes in cell proliferation. Next, when IGF2BP3 siRNA and p27 siRNA were simultaneously transfected into the cells, cell proliferation was significantly restored toward to that of mesothelioma cells transfected with only NC siRNA. This is thought to result from the suppression of p27 expression (by transfection of cells with p27 siRNA), which would otherwise be increased by transfection with IGF2BP3 siRNA. Similarly, simultaneous transfection alleviated G1 cell cycle arrest (**Figure 6B**).

Numerous studies have investigated the molecular mechanisms underlying the oncogenic function of IGF2BP3; it is associated with several regulators of cell proliferation and the cell cycle, including cyclin D1, D3, G1, and CDK6 (46, 47). *In vitro* biological analysis has shown that IGF2BP3 promotes cell proliferation and cell cycle progression from the G1 phase to S phase (48). The identification of cell cycle regulatory proteins with particularly close associations is an important clue in elucidating the metabolic mechanism of IGF2BP3.

In this study, we focused on the association between IGF2BP3 and p27. However, it is essential to note that it is not clear whether the effect of IGF2BP3 on p27 expression is due to its function as an RNA-binding protein or otherwise and whether it acts directly or indirectly. Further investigation is necessary to determine the relationship between IGF2BP3 and p27.

In summary, IGF2BP3 is involved in the proliferation of mesothelioma cells by decreasing p27 expression, which regulates the progression from the G1 phase to S phase of the cell cycle. This study highlights the potential of IGF2BP3 as a therapeutic target for the treatment of malignant mesothelioma.

## Data Availability Statement

Publicly available datasets were analyzed in this study. This data can be found here: https://www.ncbi.nlm.nih.gov/geo/query/acc.cgi?acc=GSE29370.

## Author Contributions

IE, VA, and YT designed the study. VA and YT supervised and facilitated the study IE, TN, KK, TK, and YF performed the experiments. IE analyzed the data. IE and VA interpreted the results, and IE prepared the manuscript. All authors contributed to the article and approved the submitted version.

## Conflict of Interest

The authors declare that the research was conducted in the absence of any commercial or financial relationships that could be construed as a potential conflict of interest.

## Publisher’s Note

All claims expressed in this article are solely those of the authors and do not necessarily represent those of their affiliated organizations, or those of the publisher, the editors and the reviewers. Any product that may be evaluated in this article, or claim that may be made by its manufacturer, is not guaranteed or endorsed by the publisher.

## References

[1] McDonald JC McDonald AD. The Epidemiology of Mesothelioma in Historical Context. Eur Respir J (1996) 9:1932–42. doi: 10.1183/09031936.96.09091932 8880114

[2] Myojin T Azuma K Okumura J Uchiyama I. Future Trends of Mesothelioma Mortality in Japan Based on a Risk Function. Ind Health (2012) 50:197–204. doi: 10.2486/indhealth.MS1184 22453207

[3] Zhang W Wu X Wu L Zhang W Zhao X. Advances in the Diagnosis, Treatment and Prognosis of Malignant Pleural Mesothelioma. Ann Transl Med (2015) 3:182. doi: 10.3978/j.issn.2305-5839.2015.07.03 26366399 PMC4543335

[4] Milano MT Zhang H. Malignant Pleural Mesothelioma: A Population-Based Study of Survival. J Thorac Oncol (2010) 5:1841–8. doi: 10.1097/JTO.0b013e3181f1cf2b 20975379

[5] Mancarella C Pasello M Manara MC Toracchio L Sciandra EF Picci P. Insulin-Like Growth Factor 2 mRNA-Binding Protein 3 Influences Sensitivity to Anti-IGF System Agents Through the Translational Regulation of IGF1R. Front Endocrinol (2018) 9:178. doi: 10.3389/fendo.2018.00178 PMC5919949 29731738

[6] Vikesaa J Hansen TVO Jønson L Borup R Wewer UM Christiansen J. RNA-Binding IMPs Promote Cell Adhesion and Invadopodia Formation. EMBO J (2006) 25:1456–68. doi: 10.1038/sj.emboj.7601039 PMC1440323 16541107

[7] Runge S Nielsen FC Nielsen J Lykke-Andersen J Wewer UM Christiansen J. H19 RNA Binds Four Molecules of Insulin-Like Growth Factor II mRNA-Binding Protein. J Biol Chem (2000) 275:29562–9. doi: 10.1074/jbc.M001156200 10875929

[8] Mancarella C Scotlandi K. IGF2BP3 From Physiology to Cancer: Novel Discoveries, Unsolved Issues, and Future Perspectives. Front Cell Dev Biol (2019) 7:363. doi: 10.3389/fcell.2019.00363 32010687 PMC6974587

[9] Wang T Fan L Watanabe Y McNeill PD Moulton GG Bangur C. L523S, an RNA-Binding Protein as a Potential Therapeutic Target for Lung Cancer. Br J Cancer (2003) 88:887–94. doi: 10.1038/sj.bjc.6600806 PMC2377073 12644826

[10] Pryor JG Bourne PA Yang Q Spaulding BO Scott GA Xu H. IMP-3 Is a Novel Progression Marker in Malignant Melanoma. Mod Pathol (2008) 21:431–7. doi: 10.1038/modpathol.3801016 18204432

[11] Li D Yan D Tang H Zhou C Fan J Li S. IMP3 is a Novel Prognostic Marker That Correlates With Colon Cancer Progression and Pathogenesis. Ann Surg Oncol (2009) 16:3499–506. doi: 10.1245/s10434-009-0648-5 19672661

[12] Jeng Y-M Chang C-C Hu F-C Chou H-YE Kao H-L Wang T-H. RNA-Binding Protein Insulin-Like Growth Factor II mRNA-Binding Protein 3 Expression Promotes Tumor Invasion and Predicts Early Recurrence and Poor Prognosis in Hepatocellular Carcinoma. Hepatology (2008) 48:1118–27. doi: 10.1002/hep.22459 18802962

[13] Clauditz TS Wang C-J Gontarewicz A Blessmann M Tennstedt P Borgmann K. Expression of Insulin-Like Growth Factor II mRNA-Binding Protein 3 in Squamous Cell Carcinomas of the Head and Neck. J Oral Pathol Med (2013) 42:125–32. doi: 10.1111/j.1600-0714.2012.01178.x 22643116

[14] Qian L-X Cao X Du M-Y Ma C-X Zhu H-M Peng Y. KIF18A Knockdown Reduces Proliferation, Migration, Invasion and Enhances Radiosensitivity of Esophageal Cancer. Biochem Biophys Res Commun (2021) 557:192–8. doi: 10.1016/j.bbrc.2021.04.020 33872988

[15] Samanta S Sharma VM Khan A Mercurio AM. Regulation of IMP3 by EGFR Signaling and Repression by Erβ: Implications for Triple-Negative Breast Cancer. Oncogene (2012) 31:4689–97. doi: 10.1038/onc.2011.620 PMC3337950 22266872

[16] Huang Y-Y Zhang C-M Dai Y-B Lin J-G Lin N Huang Z-X. USP11 Facilitates Colorectal Cancer Proliferation and Metastasis by Regulating IGF2BP3 Stability. Am J Transl Res (2021) 13:480–96. PMC7868846 33594305

[17] Zhang X Wang D Liu B Jin X Wang X Pan J. IMP3 Accelerates the Progression of Prostate Cancer Through Inhibiting PTEN Expression in a SMURF1-Dependent Way. J Exp Clin Cancer Res (2020) 39:190. doi: 10.1186/s13046-020-01657-0 32938489 PMC7493339

[18] Hui S Guo-Qi Z Xiao-Zhong G Chun-Rong L Yu-Fei L Dong-Liang Y. IMP3 as a Prognostic Biomarker in Patients With Malignant Peritoneal Mesothelioma. Hum Pathol (2018) 81:138–47. doi: 10.1016/j.humpath.2018.07.003 30031101

[19] Shi M Fraire AE Chu P Cornejo K Woda BA Dresser K. Oncofetal Protein IMP3, a New Diagnostic Biomarker to Distinguish Malignant Mesothelioma From Reactive Mesothelial Proliferation. Am J Surg Pathol (2011) 35:878–82. doi: 10.1097/PAS.0b013e318218985b 21566519

[20] Minato H Kurose N Fukushima M Nojima T Usuda K Sagawa M. Comparative Immunohistochemical Analysis of IMP3, GLUT1, EMA, CD146, and Desmin for Distinguishing Malignant Mesothelioma From Reactive Mesothelial Cells. Am J Clin Pathol (2014) 141:85–93. doi: 10.1309/AJCP5KNL7QTELLYI 24343741

[21] Kushitani K Amatya VJ Mawas AS Miyata Y Okada M Takeshima Y. Use of Anti-Noxa Antibody for Differential Diagnosis Between Epithelioid Mesothelioma and Reactive Mesothelial Hyperplasia. Pathobiology (2016) 83:33–40. doi: 10.1159/000442092 26735863

[22] Yoshikawa Y Sato A Tsujimura T Morinaga T Fukuoka K Yamada S. Frequent Deletion of 3p21.1 Region Carrying Semaphorin 3G and Aberrant Expression of the Genes Participating in Semaphorin Signaling in the Epithelioid Type of Malignant Mesothelioma Cells. Int J Oncol (2011) 39:1365–74. doi: 10.3892/ijo.2011.1158 21842119

[23] Nowak AK. Chemotherapy for Malignant Pleural Mesothelioma: A Review of Current Management and a Look to the Future. Ann Cardiothorac Surg (2012) 1:508–15. doi: 10.3978/j.issn.2225-319x.2012.10.05 PMC3741799 23977545

[24] Yap TA Aerts JG Popat S Fennell DA. Novel Insights Into Mesothelioma Biology and Implications for Therapy. Nat Rev Cancer (2017) 17:475–88. doi: 10.1038/nrc.2017.42 28740119

[25] Montanaro F Rosato R Gangemi M Roberti S Ricceri F Merler E. Survival of Pleural Malignant Mesothelioma in Italy: A Population-Based Study. Int J Cancer (2009) 124:201–7. doi: 10.1002/ijc.23874 18792097

[26] Terenziani R Zoppi S Fumarola C Alfieri R Bonelli M. Immunotherapeutic Approaches in Malignant Pleural Mesothelioma. Cancers (2021) 13. doi: 10.3390/cancers13112793 PMC8200040 34199722

[27] Baas P Scherpereel A Nowak AK Fujimoto N Peters S Tsao AS. First-Line Nivolumab Plus Ipilimumab in Unresectable Malignant Pleural Mesothelioma (CheckMate 743): A Multicentre, Randomised, Open-Label, Phase 3 Trial. Lancet (2021) 397:375–86. doi: 10.1016/S0140-6736(20)32714-8 33485464

[28] Zhang J Ji Q Jiao C Ren L Zhao Y Chen Y. IGF2BP3 as a Potential Tissue Marker for the Diagnosis of Esophageal High-Grade Intraepithelial Neoplasia. Onco Targets Ther (2017) 10:3861–6. doi: 10.2147/OTT.S141179 PMC5546816 28814885

[29] Sasaki M Sato Y. Insulin-Like Growth Factor II mRNA-Binding Protein 3 (IMP3) Is a Marker That Predicts Presence of Invasion in Papillary Biliary Tumors. Hum Pathol (2017) 62:152–9. doi: 10.1016/j.humpath.2016.12.028 28089541

[30] Kanzaki A Kudo M Ansai S-I Peng W-X Ishino K Yamamoto T. Insulin-Like Growth Factor 2 mRNA-Binding Protein-3 as a Marker for Distinguishing Between Cutaneous Squamous Cell Carcinoma and Keratoacanthoma. Int J Oncol (2016) 48:1007–15. doi: 10.3892/ijo.2016.3323 PMC4750532 26782292

[31] Lochhead P Imamura Y Morikawa T Kuchiba A Yamauchi M Liao X. Insulin-Like Growth Factor 2 Messenger RNA Binding Protein 3 (IGF2BP3) Is a Marker of Unfavourable Prognosis in Colorectal Cancer. Eur J Cancer (2012) 48:3405–13. doi: 10.1016/j.ejca.2012.06.021 PMC3613860 22840368

[32] Chang S Oh M-H Ji S-Y Han J Kim T-J Eom M. Practical Utility of Insulin-Like Growth Factor II mRNA-Binding Protein 3, Glucose Transporter 1, and Epithelial Membrane Antigen for Distinguishing Malignant Mesotheliomas From Benign Mesothelial Proliferations. Pathol Int (2014) 64:607–12. doi: 10.1111/pin.12216 25376377

[33] Bell JL Wächter K Mühleck B Pazaitis N Köhn M Lederer M. Insulin-Like Growth Factor 2 mRNA-Binding Proteins (IGF2BPs): Post-Transcriptional Drivers of Cancer Progression? Cell Mol Life Sci (2013) 70:2657–75. doi: 10.1007/s00018-012-1186-z PMC3708292 23069990

[34] Lederer M Bley N Schleifer C Hüttelmaier S. The Role of the Oncofetal IGF2 mRNA-Binding Protein 3 (IGF2BP3) in Cancer. Semin Cancer Biol (2014) 29:3–12. doi: 10.1016/j.semcancer.2014.07.006 25068994

[35] Sheen Y-S Liao Y-H Lin M-H Chu C-Y Ho B-Y Hsieh M-C. IMP-3 Promotes Migration and Invasion of Melanoma Cells by Modulating the Expression of HMGA2 and Predicts Poor Prognosis in Melanoma. J Invest Dermatol (2015) 135:1065–73. doi: 10.1038/jid.2014.480 25380351

[36] Jia C Tang H Yang Y Yuan S Han T Fang M. Ubiquitination of IGF2BP3 by E3 Ligase MKRN2 Regulates the Proliferation and Migration of Human Neuroblastoma SHSY5Y Cells. Biochem Biophys Res Commun (2020) 529:43–50. doi: 10.1016/j.bbrc.2020.05.112 32560817

[37] Otto T Sicinski P. Cell Cycle Proteins as Promising Targets in Cancer Therapy. Nat Rev Cancer (2017) 17:93–115. doi: 10.1038/nrc.2016.138 28127048 PMC5345933

[38] Bracken AP Ciro M Cocito A Helin K. E2F Target Genes: Unraveling the Biology. Trends Biochem Sci (2004) 29:409–17. doi: 10.1016/j.tibs.2004.06.006 15362224

[39] Polyak K Kato JY Solomon MJ Sherr CJ Massague J Roberts JM. p27Kip1, a Cyclin-Cdk Inhibitor, Links Transforming Growth Factor-Beta and Contact Inhibition to Cell Cycle Arrest. Genes Dev (1994) 8:9–22. doi: 10.1101/gad.8.1.9 8288131

[40] Sheaff RJ Groudine M Gordon M Roberts JM Clurman BE. Cyclin E-CDK2 Is a Regulator of p27Kip1. Genes Dev (1997) 11:1464–78. doi: 10.1101/gad.11.11.1464 9192873

[41] Chu I Sun J Arnaout A Kahn H Hanna W Narod S. P27 Phosphorylation by Src Regulates Inhibition of Cyclin E-Cdk2. Cell (2007) 128:281–94. doi: 10.1016/j.cell.2006.11.049 PMC1961623 17254967

[42] Chu C Geng Y Zhou Y Sicinski P. Cyclin E in Normal Physiology and Disease States. Trends Cell Biol (2021) 31:732–46. doi: 10.1016/j.tcb.2021.05.001 PMC8364501 34052101

[43] Inoshita S Terada Y Nakashima O Kuwahara M Sasaki S Marumo F. Regulation of the G1/S Transition Phase in Mesangial Cells by E2F1. Kidney Int (1999) 56:1238–41. doi: 10.1046/j.1523-1755.1999.00705.x 10504464

[44] Koff A Giordano A Desai D Yamashita K Harper JW Elledge S. Formation and Activation of a Cyclin E-Cdk2 Complex During the G1 Phase of the Human Cell Cycle. Science (1992) 257:1689–94. doi: 10.1126/science.1388288 1388288

[45] Lee G-E Lee C-J An H-J Kang HC Lee HS Lee JY. Fargesin Inhibits EGF-Induced Cell Transformation and Colon Cancer Cell Growth by Suppression of CDK2/Cyclin E Signaling Pathway. Int J Mol Sci (2021) 22. doi: 10.3390/ijms22042073 PMC7922630 33669811

[46] Rivera Vargas T Boudoukha S Simon A Souidi M Cuvellier S Pinna G. Post-Transcriptional Regulation of Cyclins D1, D3 and G1 and Proliferation of Human Cancer Cells Depend on IMP-3 Nuclear Localization. Oncogene (2014) 33:2866–75. doi: 10.1038/onc.2013.252 23812426

[47] Palanichamy JK Tran TM Howard JM Contreras JR Fernando TR Sterne-Weiler T. RNA-Binding Protein IGF2BP3 Targeting of Oncogenic Transcripts Promotes Hematopoietic Progenitor Proliferation. J Clin Invest (2016) 126:1495–511. doi: 10.1172/JCI80046 PMC4811152 26974154

[48] Huang W Li Y Zhang C Zha H Zhou X Fu B. IGF2BP3 Facilitates Cell Proliferation and Tumorigenesis *via* Modulation of JAK/STAT Signalling Pathway in Human Bladder Cancer. J Cell Mol Med (2020) 24:13949–60. doi: 10.1111/jcmm.16003 PMC7753985 33094561

