# Supplementary material for: Insulin-Like Growth Factor 2 mRNA Binding Protein 3 Promotes Cell Proliferation of Malignant Mesothelioma Cells by Downregulating p27Kip1
Source: Front Oncol. 2022 Jan 19;11:795467. doi: 10.3389/fonc.2021.795467 (PMC8807558; doi:10.3389/fonc.2021.795467)
Supplement: Supplementary file 1 [file DataSheet_1.pdf]

Supplementary Figure.

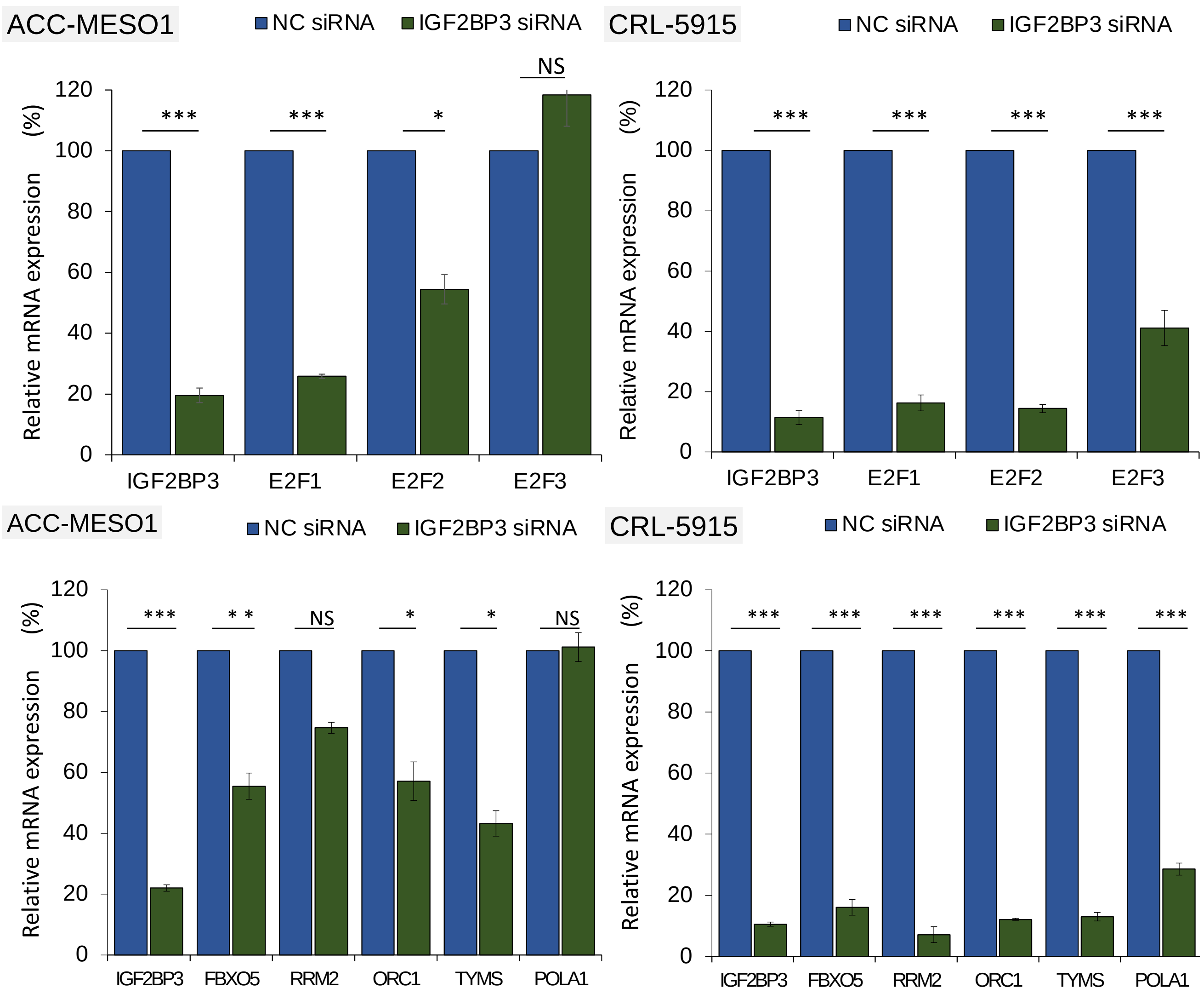

Figure Legend

E2F family and E2F target genes mRNA expression in IGF2BP3 knockdown. Upper panel: E2F1, 2, 3 mRNA expression in IGF2BP3 knockdown. In ACC-MESO1 and CRL-5915, knockdown of IGF2BP3 significantly downregulates E2F1, E2F2, and E2F3 expression in ACC-MESO1 and CRL-5915 except for E2F3 expression in ACC-MESO1. Lower panel: FBXO5, RRM2, ORC1, TYMS, and POLA1 mRNA expression in IGF2BP3 knockdown. FBXO5, RRM2, ORC1, TYMS, and POLA1 are selected as the E2F target genes using the pathway browser of [Reactome website]. In ACC-MESO1 and CRL-5915, knockdown of IGF2BP3 significantly suppresses the expression of TYMS, POLA1, ORC1, FBXO5, and RRM2 mRNAs, except for RRM2 and POLA1 in ACC-MESO1.

NC: negative control; NS: no significance; *: $p < 0.05$; **: $p < 0.01$; ***: $p < 0.001$

Supplementary Table. List of primers used for RT PCR of E2F target genes

Gene	Forward primer	Reverse primer
E2F1	CGGCGCATCTATGACATCAC	GTCAACCCCTCAAGCCGTC
E2F2	CTCTCTGAGCTTCAAGCACCTG	CTTGACGGCAATCACTGTCTGC
E2F3	AGCGGTCATCAGTACCTCTCAG	TGGTGAGCAGACCAAGAGACGT
FBXO5	GCTGTCATGTATTGGGTCA	GTCTACTGGTCTCTAGTGCTTCT
RRM2	CACGGAGCCGAAAACTAAAGC	TCTGCCTTCTTATACATCTGCCA
ORC1	ACTACCCCACAAGGCTGAAGA	AGTGCAGTTTTCGATCCAACA
TYMS	CTGCTGACAACCAAACGTGTG	GCATCCCAGATTTTCACTCCCTT
POLA1	ACGCCAGGATGATGACTGGA	GTCACTGCGAGCTTCTTTACAT
